# Ten-year follow up of cosmetic outcome, overall survival, and disease-free survival in endoscope-assisted partial mastectomy with filling of dead space using absorbable mesh for stage ≤ IIA breast cancer: comparison with conventional conservative method

**DOI:** 10.1186/s12905-021-01399-x

**Published:** 2021-06-24

**Authors:** Nobuyuki Takemoto, Ai Koyanagi, Hiroshi Yamamoto

**Affiliations:** 1 Department of Breast and Thyroid Surgery, Japan Medical Alliance East Saitama General Hospital, 5-517, Yoshino, Satte-City, Saitama-Pref 340-0153 Japan; 2 Research and Development Unit, Parc Sanitari Sant Joan de Déu, Fundació Sant Joan de Déu, CIBERSAM, Sant Boi de Llobregat, Barcelona Spain; 3 ICREA, Pg. Lluis Companys 23, Barcelona, Spain

**Keywords:** Breast cancer, Endoscopic surgery, Cosmetic outcome, Overall survival, Disease-free survival, Tumor location

## Abstract

**Background:**

Data on long-term cosmetic outcome, overall survival, and disease-free survival of endoscope-assisted partial mastectomy (EAPM) for breast cancer are scarce. Thus, we examined these outcomes after a 10-year follow-up period, and compared with conventional conservative method (CCM).

**Patients and methods:**

Data on 257 patients with stage ≤ IIA breast cancer who underwent CCM (n = 125) or EAPM (n = 132) were analyzed. Cosmetic outcome at 2, 5, and 10 years was evaluated by 5 criteria (breast retraction assessment, nipple deviation, atrophy, skin change, scar). For overall mortality, breast cancer-specific mortality, and recurrence, the risk by operation method was tested by Cox proportional hazard models.

**Results:**

EAPM performed significantly better than CCM in terms of cosmetic outcomes for location B at 2, 5, and 10 year-follow ups. As for cosmetic outcomes by individual criteria, EAPM had significantly higher proportions of satisfactory results for scar across all follow-up periods, and atrophy at 2-year and 10-year follow-up. There were no significant differences in terms of overall mortality, breast cancer-specific mortality, and recurrence between EAPM and CCM. The rates of patients who experienced local recurrence were similar between CCM and EAPM.

**Conclusion:**

EAPM is better than CCM in terms of long-term cosmetic outcome, especially for location B. As a surgical treatment for breast cancer, EAPM is comparable to CCM in terms of mortality and recurrence.

## Introduction

With the development of oncoplastic surgery, the indications for breast conservative therapy (BCT) may have become more restrictive, but BCT still plays an important role in the surgical treatment of early breast cancer. BCT is an excellent technique that can achieve the same survival rate as total mastectomy with a small resection, but the breast volume is always reduced unless some compensation is applied to the resection area. Various methods have been tried so far, such as pedicle and free flaps [1], fat injection [2], and breast implants [1] in order to prevent the decrease in volume and to maintain the cosmetic outcome. Currently, there are two techniques for BCT: conventional conservative method (CCM) and endoscopic surgery. In 2003, we introduced endoscope-assisted partial mastectomy (EAPM) in our institute, which uses an endoscope as an aid and fills the resection area with an absorbable mesh. In 2012, we published an article [3] comparing the post-operative cosmetic outcome between the two methods, and showed that EAPM is superior in terms of cosmetic outcome, especially in location B, and that atrophy and scar are significantly less. However, this previous study had a short follow-up period and could not assess changes over time, while it was also not possible to assess important clinical outcomes such as overall survival (OS) and disease-free survival (DFS) due to too few events. In particular, since EAPM consists of skin-sparing partial mastectomy (SSPM), which may potentially increase risk of local recurrence [4, 5], it is of vital importance to assess this outcome. Thus, using data on EAPM that has accumulated over 18 years in our institute, we conducted a follow-up study of the previous study [3] and aimed to examine the long-term clinical outcomes of EAPM. Specifically, the aims of the current study were to compare: (a) post-operative cosmetic outcome at 2, 5, and 10-year follow-up between CCM and EAPM; and (b) post-operative 10-year OS and DFS between CCM and EAPM. To the best of our knowledge, this is the first study that describes long-term outcomes of EAPM including cosmetic outcomes.

## Materials and methods

Patients with stage ≤ IIA primary breast cancer who underwent BCT between May 1997 and October 2010 were retrospectively reviewed. Indication for BCT was based on the guidelines of BCT issued by the Japanese Breast Cancer Society [6]. Patients with primary tumor size > 3 cm, multicentric breast cancer and tumors fixed to the skin or muscle were not subject to BCT. Furthermore, patients who underwent BCT after receiving neoadjuvant chemotherapy were excluded from this study because the risk of intramammary local recurrence is not the same as in those who did not undergo neoadjuvant chemotherapy. All of these findings were confirmed by pre-operative examinations (mammography (MMG), ultrasound (US), enhanced multi-detector-row computer tomography (MDCT), enhanced magnetic resonance imaging etc.). Tumor location was determined as the breast area that occupies > 70% of the tumor area as evidenced by MDCT. Tumor location was defined as: A, upper-inner quadrant; B, lower-inner quadrant; C, upper-outer quadrant; D, lower-outer quadrant. If the primarily involved site could not be determined due to equal extent of involvement in 2 or more areas, tumor location was expressed as the predominant area that occupied the resection area.

EAPM was introduced as the first-line therapy in September 2003 after approval by the ethics committee in East Saitama General Hospital. Details on the procedures of CCM and EAPM [3, 7] are provided in the “Appendix”. Detailed information on both CCM and EAPM methods was provided to all patients and family members pre-operatively, and informed consent was obtained from all patients. EAPM was only performed if the patient agreed to undergo this operation, and ordinary CCM was performed otherwise. In our study, 42 women declined EAPM despite availability of EAPM and underwent CCM. All EAPM was performed by a single operator and all CCM was operated or assisted by that same operator.

We did not examine the surgical margin of the resected breast tissue histologically by frozen section during operation, but post-operative histopathological examination was performed on the resected specimen to confirm the diagnosis. In three cases (CCM 2 cases, EAPM 1 case) where the cancer cells were exposed to the surface of the pathological specimen, salvage operation (total mastectomy) was added, and such cases were excluded from this study.

Further examinations such as estrogen and progesterone receptors, HER2 score and ki67 were also conducted. Sentinel lymph node (SLN) [8] biopsy was performed for clinically N0 patients. If SLN was positive, axillary incision was extended by 1–2 cm and axillary dissection was added. In cases where N1 was clearly suspected based on pre-operative examinations, axillary lymph nodes dissection was conducted from the beginning. Furthermore, in this current study, only cases that underwent BCT with separate incisions (i.e., incision on the breast for tumor resection and an axillary incision for SLN biopsy or axillary lymph nodes dissection) were reviewed.

All patients in the study underwent tangential field irradiation of the whole breast (50 Gy), with a dose per fraction of 2 Gy in 5 weeks. If pathological margins were close to the tumor edge (≤ 5 mm), a boost of 10 Gy or tumor bed 60 Gy was added and patients were not subject to subsequent operation. Indication for post-operative chemotherapy and hormone therapy was based on the guidelines for breast cancer [9]. The first line regimen of chemotherapy was Anthracycline regimen (AC: doxorubicin and cyclophosphamide, EC: epirubicin and cyclophosphamide, FEC: fluorouracil, epirubicin and cyclophosphamide), and according to patient status and pathological findings, taxan regimen was added and classical CMF (cyclophosphamide, methotrexate and fluorouracil), TC (docetaxel and cyclophosphamide) and trastuzumab were also selected. First line hormonal therapy was tamoxifen (TAM) or TAM plus goserelin (premenopausal patients), and TAM or Aromatase Inhibitor (patients in menopausal status for more than 5 years).

### Objective cosmetic assessment

As in a previous publication [3], objective cosmetic assessment was based on the method described by Al-Ghazal et al. [10]. The score consists of 5 criteria (breast retraction assessment (BRA) [11], nipple deviation (ND) [12], atrophy, skin change, and scar) with each criterion ranging from 0 to 2 with higher scores corresponding to better cosmetic outcome. BRA assessed the lack of symmetry between the nipple positions and was calculated by the method described by Pezner et al. [11] BRA was scored as 2 for < 3.1 cm, 1 for 3.1–6.5 cm, and 0 for > 6.5 cm. ND was calculated as the percent difference between the anterior breast surface length from the sternal notch to the nipple on the affected side and that on the healthy side, with the patient in an erect position using the method described by Noguchi et al. [12] The degree of ND was scored as 2 for < 5%, 1 for 5% to 10%, 0 for > 10%. The degree of breast atrophy was scored as 2 for not atrophic, 1 for slightly atrophic, and 0 for atrophic. Skin change was based on the level of edema, pigmentation, and telangiectasia and scored as 2 for no change, 1 for slight, and 0 for severe change. The degree of surgical scar was based on the visibility from anterior view and was scored as 2 for not visible, 1 for slightly visible, and 0 for remarkable. The cosmetic outcome was assessed by summing these scores, and total scores of ≤ 6 and ≥ 7 were considered unsatisfactory and satisfactory, respectively. We also performed individual analyses by the five criteria where we considered score 2 as satisfactory, and 0 or 1 as unsatisfactory. Assessment of cosmetic outcome was conducted at 3 timepoints (i.e., at 2, 5, and 10-year follow-up) by a single individual.

### Post-operative follow-up

Routine follow-up consisted of physical examination at 3-month interval, blood test including tumor markers (CEA, NCC-ST439, CA15-3) and US every 6 months, and yearly MMG for the first 5 years after the operation. For the first 5–10 years, image examination (e.g., CT) was carried out at one-year interval. More frequent image examinations were performed only when indicated by symptoms or findings on physical examination or when recurrence risk was deemed to be high. Dates of death due to any cause, death due to breast cancer, and identification of first recurrence were recorded.

### Statistical analysis

Statistical analysis was conducted by Stat Mate IV (Microsoft Excel, statistically soft, ATMS, Co. Ltd, Tokyo, Japan) and Stata 14.2 (Stata Corp LP, College station, Texas, USA). Student’s *t*-tests and Chi-squared tests were used to test differences in patient characteristics by method of operation, for continuous and categorical variables, respectively. Next, in samples stratified by tumor location, we compared the proportion of patients with satisfactory cosmetic results (i.e., total score ≥ 7 by method described by Al-Ghazal et al. [10]) between CCM and EAPM at 2, 5, and 10-year follow-up with the use of Chi-squared tests unless there were cells with less than 5 observations, in which case Fisher’s exact text was used. Finally, the proportion of patients with satisfactory cosmetic results based on each individual criterion (i.e., score ≥ 2) at 2, 5, and 10-year follow-up were also compared between the two methods of operation with the use of Chi-squared tests.

Cox proportional hazard models were constructed to estimate the risk for overall mortality, breast cancer-specific mortality, and recurrence as a function of the surgical method (i.e., CCM or EAPM), while adjusting for age, boost, chemotherapy, hormone therapy, n factor, and t factor. The time to onset of these outcomes was calculated and the start of the risk period was date of operation. The censoring date was the end of follow-up or death, whichever came first for mortality. For the analysis on breast-cancer specific mortality, individuals who died of other causes were censored on their date of death. For recurrence, the censoring date was the end of follow-up, death, or first identification of recurrence. Kaplan–Meier survival curves were drawn to graphically display the risk for overall mortality, breast cancer-specific mortality, and recurrence by operation method. Results of the Cox proportional hazard models are reported as hazard ratios (HRs) with 95% confidence intervals (CIs). The level of statistical significance was set at *p* < 0.05.

## Results

All the patients were women and the mean (SD) age was 56.3 (11.7) years. The CCM and EAPM groups consisted of 125 and 132 patients, respectively. Patient characteristics by method of operation are shown in Table 1. Patients who underwent EAPM were significantly younger, more likely to be T0, T2, and N0, while operation time in EAPM was significantly longer with more bleeding.

**Table1:** Patient characteristics by method of operation

Characteristics	CCM (n = 125)	EAPM (n = 132)	*P* value^a^
Age (year)	58.4 (11.0)	54.1 (12.4)	0.003
BMI (kg/m^2^)	22.9 (3.9)	23.3 (4.3)	0.358
Margin positive (≤ 5 mm) and Boost	20.0%	29.6%	0.077
Chemotherapy	38.4%	47.7%	0.131
Hormone therapy	72.0%	70.5%	0.784
Tumor location^b^			
A	20.0%	22.0%	0.907
B	16.0%	16.7%	
C	46.4%	47.0%	
D	17.6%	14.4%	
T factor^c^			
0	4.0%	5.3%	0.081
1	86.4%	75.8%	
2	9.6%	18.9%	
N factor^c^			
0	79.2%	90.9%	0.008
1	20.8%	9.1%	
Stage^c^			
0	3.2%	5.3%	0.698
1	68.0%	67.4%	
2a	28.8%	27.3%	
Operation time (min)	62.0 (25.4)	79.8 (23.5)	< 0.001
Operative bleeding (mL)	52.0 (47.5)	98.6 (84.1)	< 0.001

*CCM* conventional conservative method, *EAPM* endoscope-assisted partial mastectomy, *BMI* body mass index

Data are column % or mean (standard deviation)

^a^*P* value was calculated by χ^2^ test for categorical variables and Student’s *t*-tests for continuous variables

^b^Tumor location was defined as; A (upper inner quadrant); B (lower inner quadrant); C (upper outer quadrant); D (lower outer quadrant)

^c^Based on TNM classification of malignant tumors (14)

**Fig. 1:**
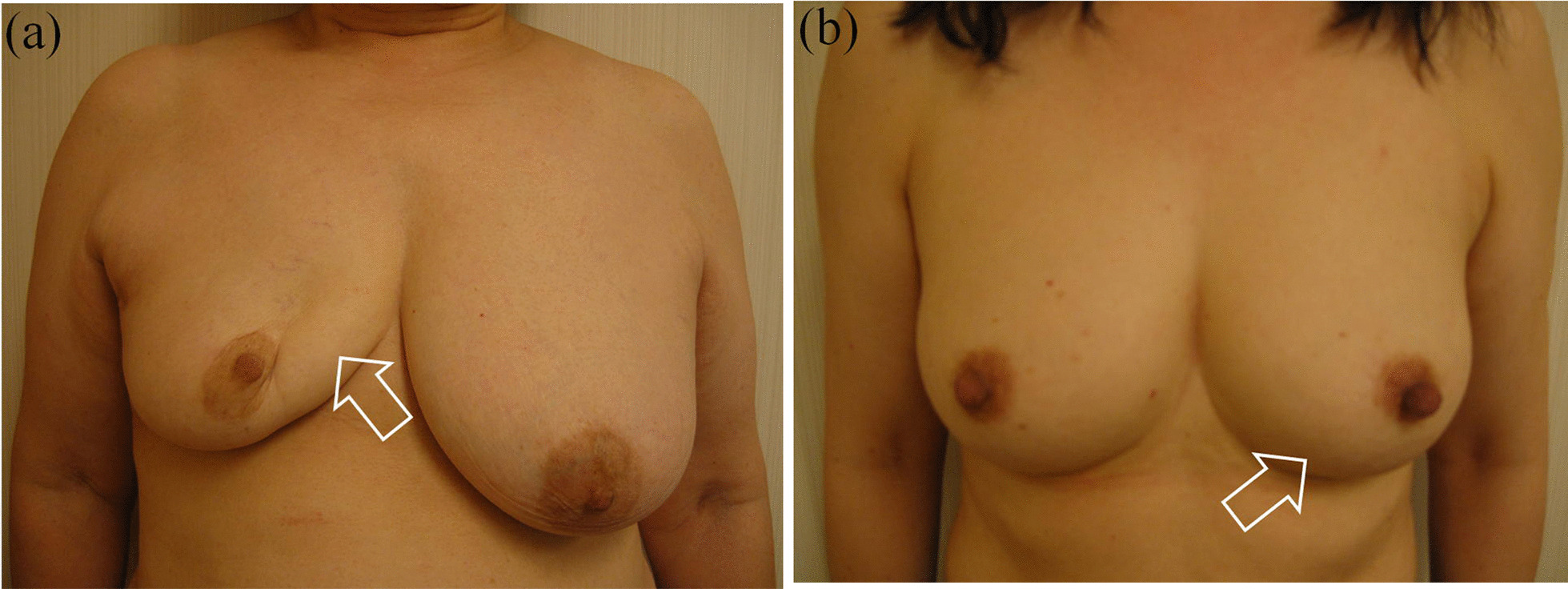
Examples of **a** unsatisfactory and **b** satisfactory cosmetic outcome. **a** Lesion in right location A: Remarkable atrophy of location A, decrease in breast retraction assessment, and strong nipple retraction. **b** Lesion in left location B: Almost symmetrical, no atrophy, and no decrease in breast retraction assessment

In terms of cosmetic outcome by location, across all follow-up periods, we found that the proportion of patients with satisfactory results was significantly higher in EAPM (vs. CCM) only for location B (Table 2). In the overall sample with all locations combined, EAPM had significantly higher proportion of satisfactory results at two-year follow-up but not in subsequent follow-ups. Next, as for cosmetic outcomes by individual criteria, we found that EAPM had significantly higher proportions of satisfactory results for scar across all follow-up periods, and atrophy at 2-year and 10-year follow-up (Table 3). Examples of unsatisfactory and satisfactory cosmetic results are illustrated in Fig. 1. The case with unsatisfactory results shows remarkable atrophy, strong nipple retraction etc., whereas the case with satisfactory results shows an almost intact breast.

**Table 2:** Percentage of satisfactory cosmetic results by each tumor location and method of operation at 2, 5, and 10 years of post-operative follow-up

Follow-up	Location^b^	CCM	EAPM	*P* value^a^
2nd year	A	64.0% (16/25)	75.9% (22/29)	0.341
	B	21.1% (4/19)	68.2% (15/22)	0.004
	C	71.9% (41/57)	79.0% (49/62)	0.367
	D	22.7% (5/22)	42.1% (8/19)	0.184
	Total	53.7% (66/123)	71.2% (94/132)	0.004
5th year	A	63.6% (14/22)	51.7% (15/29)	0.395
	B	21.1% (4/19)	63.6% (14/22)	0.011
	C	71.9% (41/57)	73.7% (42/57)	0.833
	D	26.3% (5/19)	37.5% (6/16)	0.478
	Total	54.7% (64/117)	62.1% (77/124)	0.244
10th year	A	61.9% (13/21)	50.0% (14/28)	0.407
	B	17.7% (3/17)	60.0% (12/18)	0.018
	C	71.2% (37/52)	73.6% (39/53)	0.781
	D	27.8% (5/18)	42.9% (6/14)	0.373
	Total	53.7% (58/108)	61.7% (71/115)	0.225

*CCM* conventional conservative method, *EAPM* endoscope-assisted partial mastectomy

Satisfactory results refer to total score ≥ 7 by method described by Al-Ghazal et al. [11]

^a^*P* value was calculated by χ^2^ test unless there were < 5 observations per cell in which case, Fisher’s exact test was used

^b^Tumor location was defined as; A (upper inner quadrant); B (lower inner quadrant); C (upper outer quadrant); D (lower outer quadrant)

**Table 3:** Percentage of satisfactory results by five individual criteria and method of operation at 2, 5, and 10 years of post-operative follow-up

Follow-up	Criterion	CCM	EAPM	*P* value^a^
2nd year	BRA	80.5% (99/123)	76.5% (101/132)	0.441
	ND	52.0% (64/123)	58.3% (77/132)	0.312
	Atrophy	34.2% (42/123)	54.5% (72/132)	0.001
	Skin change*	37.4% (46/123)	46.2% (61/132)	0.154
	Scar	33.3% (41/123)	56.1% (74/132)	< 0.001
5th year	BRA	81.2% (95/117)	75.0% (93/124)	0.246
	ND	50.4% (59/117)	54.0% (67/124)	0.575
	Atrophy	32.5% (38/117)	44.4% (55/124)	0.058
	Skin change*	38.5% (45/117)	47.6% (59/124)	0.153
	Scar	34.2% (40/117)	55.7% (69/124)	0.001
10th year	BRA	80.6% (87/108)	74.8% (86/115)	0.302
	ND	50.0% (54/108)	52.2% (60/115)	0.746
	Atrophy	30.6% (33/108)	44.4% (51/115)	0.034
	Skin change*	40.7% (44/108)	47.0% (54/115)	0.350
	Scar	30.6% (33/108)	55.7% (64/115)	< 0.001

*CCM* conventional conservative method, *EAPM* endoscope-assisted partial mastectomy, *BRA* breast retraction assessment, *ND* nipple deviation

Satisfactory results refer to score 2 for each scoring criterion described by Al-Ghazal et al. [10]

^a^*P* value calculated by χ^2^ test

*Edema, pigmentation, telangectasia

**Fig. 2:**
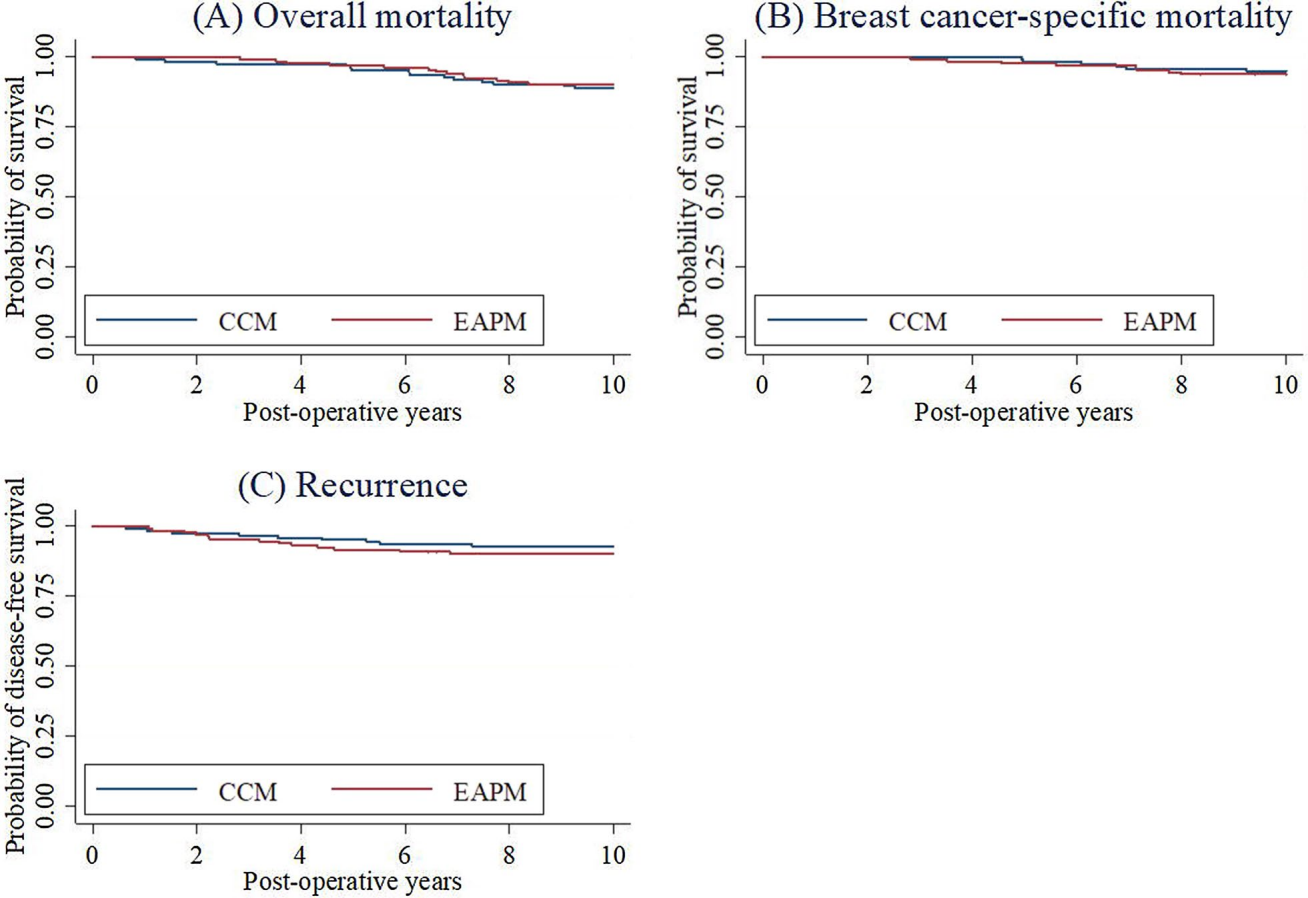
Kaplan–Meier survival curves of postoperative **a** overall mortality, **b** breast cancer-specific mortality, and **c** recurrence by conventional conservative method (CCM) and endoscope-assisted partial mastectomy (EAPM)

The Kaplan–Meier survival curves for overall mortality, breast cancer-specific mortality, and recurrence are shown in Fig. 2. The 10-year overall survival rate was 88.8% for CCM and 90.1% for EAPM, and the corresponding figures for breast cancer-specific 10-year survival rate were 95.0% and 93.8%, respectively, while those for 10-year DFS were 92.6% and 90.1%, respectively. The results of the Cox proportional hazard models showed that EAPM was not associated with a significantly higher risk for overall mortality (HR = 1.06; 95%CI = 0.48–2.37; *P* = 0.879), breast cancer-specific mortality (HR = 1.72; 95%CI = 0.54–5.52; *P* = 0.360), or recurrence (HR = 1.61; 95%CI = 0.65–4.02; *P* = 0.308) (data shown only in text).

Finally, in terms of first site of recurrence, we did not find any evidence of significant differences between CCM and EAPM. Specifically, the rates of patients who experienced local recurrence (i.e., residual mammary gland, isolated skin and subcutaneous recurrence) were similar between CCM (3.2%) and EAPM (3.0%). Of note, there were 25 cases where the surgical margin was ≤ 5 mm in CCM and 39 cases in EAPM, but no local recurrence was found in these cases. Recurrence in the form of distant metastasis (i.e., any recurrence in distant organs and lymph node metastasis except the axilla) was more common in EAPM (6.8%) than in CCM (3.2%) but this difference was not statistically significant (Chi-squared test *P* = 0.299).

## Discussion

In our study including 257 women with stage ≤ 2A breast cancer who underwent CCM or EAPM and followed for up to 10 years, we found that EAPM performed significantly better than CCM in terms of cosmetic outcomes for location B at 2, 5, and 10 year-follow ups. Overall, the proportion of satisfactory cosmetic outcomes was significantly higher in EAPM than in CCM at 2-year follow-up but this was non-significant in subsequent follow-ups. Furthermore, in terms of the individual criteria of cosmetic outcomes, we found that EAPM was significantly superior to CCM in terms of atrophy and scar and this effect was consistently shown across all follow-ups (i.e., 2, 5, and 10 years), although atrophy did not reach statistical significance at 5-year follow-up (*P* = 0.058). Finally, survival analysis showed that there were no significant differences in terms of post-operative overall mortality, breast cancer-specific mortality, and recurrence between EAPM and CCM.

The finding that better cosmetic outcome overall in EAPM was only found at 2-year follow-up may be related to the filling of the dead space with an absorbable mesh. In EAPM, the dead space is filled with absorbable meshes plus reactive tissue fluid, two to three days after the injection of Vicryl mesh wrapped in Intershied. Foreign-body reaction occurs and promotes granulation and the formation of connective tissue along the margin of the dead space. One to two months post-operatively, the margin becomes suitably hard, and the dead space forms a comparatively fixed shape [13, 14]. It is possible that the benefits of EAPM decreased after two years due to reduction in the fluid stored in the dead space, which may have led to asymmetry via traction of the surrounding tissue. It is difficult to clarify the reason for the fluid loss because pathological research cannot be performed. However, one of the causes may be insufficient margin formation (i.e., encapsulation), which plays an important role in the cosmetic outcome of EAPM.

Margin formation may also be affected by factors such as age, menopause status, BMI, tumor location, excision volume, the ease of granulation tissue formation, and mammary gland shape, as well as other external factors such as chemotherapy, endocrine therapy, and radiation therapy. These conditions are all different in each case, and thus, it is unknown which factor had the greatest impact on cosmetic outcome, especially considering that these factors may be intertwined and have synergistic effects. However, based on our results, in the case of EAPM, it seems to be clear that tumor location is a determinant of cosmetic outcome given that location B was significantly and consistently associated with approximately 3 times higher rates of satisfactory results in EAPM compared to CCM during the 10-year follow-up.

It is widely known that a lower tumor location is associated with postoperative cosmetic outcome difficulties in BCT [15-17]. However, EAPM performed much better than CCM in location B and this effect was long-lasting (i.e., observed for up to at least 10 years after the operation). This may be related to the fact that cases of location B usually have a smaller resected volume than in those of other locations in EAPM, and it may be easily fixed with the use of a brassiere after operation. Therefore, margin formation may have completed at an early stage with early stabilization of cosmetic outcomes. As for upper tumor location, although there were no significant differences in terms of cosmetic outcomes between EAPM and CCM across the years, when focusing only on EAPM, we found some difference between location A and C in terms of long-term outcomes. Specifically, of those who had overall satisfactory results at 2-year follow up, 33.3% (7/22) and 10.6% (5/47) had unsatisfactory results at 5-year follow up for location A and C, respectively, suggesting that location A is associated with worsening in cosmetic outcomes across time. A further examination into the individual criteria showed that a change from score 2 (satisfactory based on individual criteria) to score 0 or 1 (unsatisfactory) between 2 and 5-year follow-up in EAPM cases of location A and C occurred mainly for atrophy (A: 20.0%; n = 3/15, C: 20.0%; n = 7/35), ND (A: 11.1%; n = 2/18, C: 5.7%; n = 2/35), and BRA (A: 16.7%; n = 4/24, C: 2.0%; n = 1/49). As shown above, among patients who scored 2 for BRA at 2-year follow-up, a much larger proportion scored 0 or 1 at 5-year follow-up in location A as compared to location C. This may indicate that in location A, when the fluid in the dead space decreases, the accompanying traction of surrounding tissue is likely to affect the cosmetic outcome, but in location C, this effect may be minimal. However, given that this observation is derived from a small number of cases, this needs to be confirmed with larger studies. For the moment, we only consider this to be a possible characteristic associated with tumor location in EAPM. Finally, the fact that EAPM was superior to CCM in terms of scar and atrophy up to at least 10-years follow-up is likely to be explained by the periareolar incision where the scar is less noticeable, and the placing of buried subcutaneous sutures using 5-0 PDS-2 clear (see “Appendix” for details), while the insertion of the mesh may have led to less atrophy for certain locations (i.e., B and C).

There was no significant difference between CCM and EAPM in terms of all-cause mortality, breast cancer-specific mortality, and recurrence. Furthermore, we found no evidence of difference in the form of recurrence between the two procedures. Despite the fact that SSPM is associated with higher risk of local recurrence [4, 5], EAPM did not significantly increase local recurrence. We believe that this may be because (1) EAPM is not indicated for tumor size of > 3 cm, and cases with tumors fixed to the skin or muscle, and (2) a boost of 10 Gy or tumor bed 60 Gy was added if pathological margins were close to the tumor edge (≤ 5 mm). Fan et al. [18] reported that the indication of endoscopic subcutaneous mastectomy is limited to a distance of more than 0.5 cm between the tumor surface and skin, determined by preoperative US, and our indication was similar. In addition, we judged that cases with cancer cells within 5 mm from the surface of the resected breast tissue were margin positive and added boost to all these cases. However, this is a criterion that is too strict in view of the current world trends [19]. We believe that this strict measure may have contributed to the reduction of local recurrence, but the possibility of overtreatment cannot be ruled out, and further studies are needed.

The results of our study showed that the beneficial effects of EAPM in terms of cosmetic outcomes is long-lasting but may be mainly restricted to location B. Whether there are any methods to improve cosmetic outcomes of EAPM for other locations is an area for future research. For example, given that one report suggested that the cosmetic outcome can be restored by injecting saline into the dead space when the fluid inside the dead space decreases [20], we attempted to inject saline under the guide of US at the 5th year in 5 EAPM cases of A and C areas which had experienced worsening of cosmetic outcome due to volume loss. However, in all cases, there was a complaint of strong pain before the injection volume exceeded 20 ml, and therefore, the procedure had to be interrupted. In addition, intraoperative US of these cases did not show clear stretching of the margin wall even during the injection of saline, and only showed that the entire dead space cavity swelled very slightly, while improvement in cosmetic outcome was not observed in any of these cases. Concrete conclusions cannot be drawn based on our experience of only 5 cases, but considering that cosmetic evaluation remained unchanged between 5 and 10 years, it is highly likely that the margin wall is basically fixed by this time. Our experience shows that at least after 5 years, it may be difficult to improve the cosmetic outcome by injecting additional saline, but there remains the possibility that this injection method may be beneficial at earlier timepoints.

It is also important to note that mesh infection is a complication of EAPM which may negatively affect cosmetic outcomes [3]. We implemented several countermeasures, but the rate of mesh infection did not change significantly. Currently, there are many operations that use absorbable meshes [21, 22], however, in EAPM, the mesh is placed directly in the dead space under the skin, and the reactive tissue fluid stored in the limited space may increase risk of infection.

Finally, a limitation of this study is that it was a single-center experience with relatively small sample size. Similar studies with larger sample size should be conducted to confirm our findings.

## Conclusion

EAPM is better than CCM in terms of long-term cosmetic outcome, especially for location B. As a surgical treatment for breast cancer, EAPM is comparable to CCM in terms of OS and DFS. However, it should be noted that there are some complications specific to EAPM, while it is also important to avoid cases where the tumor is close to the skin or a sufficient margin for EAPM cannot be secured.

## Data Availability

The datasets used or analyzed during the current study are available from the corresponding author on reasonable request.

## Appendix

In CCM, for tumors of lateral location, an approximately 10 cm oblique incision was placed in the axillary region, and wide excision was performed. For tumors of medial location, wide excision was performed under the oblique incision on the tumor. The surgical wound was closed by placing subcutaneous sutures using 3-0 Vicryl (Ethicon Endo-Surgery Co.) and 4-0 Nylon was applied to the skin surface.

In EAPM, the tumor was confirmed by means of ultrasonography, and a mixture of a small amount of indigo carmine (Daiichi-Sankyo, Tokyo, Japan) and xylocaine jelly 2% (2–3 mL) (AstraZeneca; Tokyo, Japan) were injected to mark the resection line of the mammary gland at 12 point locations under ultrasound guidance, where the resection line was delineated further than 2.1 cm from the edge of the tumor. The periareolar incision was placed at about 120°. From the incision, a Visiport™ Plus Optical Trocar (Covidien; Mansfi eld, MA, USA) with a rigid endoscope was inserted to create a 160°-wide subcutaneous tunnel. The light at the tip of the Visiport™ was used to confirm that the tip was inserted parallel to the skin and the breast tissue was being detached. Using PowerStar Bipolar Scissors (Ethicon; Somerville, NJ, USA) or Harmonic scalpel (Ethicon Endo-Surgery; Cincinnati, OH, USA), the tissue between the tunnels was cut, and a skin flap with a slight amount of fat was created. A vertical incision was placed at the marking point closest to the nipple, using an electrocautery to expose and cut the pectoralis major fascia. Between the fascia and the muscle, a Round-Preperitoneal Distention Balloon (PDB) (Covidien; Mansfi eld, MA, USA) was inserted, and the mammary gland was separated from the pectoralis major muscle. The balloon was inflated for 5 min and this also served for hemostasis. Then, the location of the tumor was reconfirmed by palpation. Using an electrocautery, the mammary gland was cut vertically while connecting the marking points, so that the excised tumor was a cylindrical mass. After thorough hemostasis, the mastectomized area was slightly sutured without inducing breast deformation. Vicryl® mesh (15 × 15 cm, Ethicon; Somerville, NJ, USA) wrapped in Gynecare Interceed™ (7.6 × 10.2 cm; Ethicon) was used to fill the remaining dead space. The number of meshes depended on the resection volume. The surgical wound was closed by placing subcutaneous buried sutures using 5-0 PDS-II clear (Ethicon EndoSurgery; Cincinnati, OH, USA). As for axillar lymph nodes in both procedures, SLN biopsy or axillary lymph node dissection were performed.

## Abbreviations

BCT: breast conservative therapy
BMI: body mass index
OS: overall survival rate
DFS: disease-free survival rate
CCM: conventional conservative method
EAPM: endoscope-assisted partial mastectomy
SSPM: skin-sparing partial mastectomy
MMG: mammography
US: ultrasound
MDCT: enhanced multi-detector-row computer tomography
BRA: breast retraction assessment
ND: nipple deviation
